# Baseline and stress-induced levels of corticosterone in male and female Afrotropical and European temperate stonechats during breeding

**DOI:** 10.1186/s12862-017-0960-9

**Published:** 2017-05-22

**Authors:** Beate Apfelbeck, Barbara Helm, Juan Carlos Illera, Kim G. Mortega, Patrick Smiddy, Neil P. Evans

**Affiliations:** 1 Institute of Biodiversity, Animal Health and Comparative Medicine, University of Glasgow, Glasgow, Scotland G12 8QQ UK; 2 Terrestrial Ecology Research Group, Department of Ecology and Ecosystemmanagement, Technische Universität München, School of Life Sciences Weihenstephan, D-85354 Freising, Germany; 3 Department of Migration and Immunoecology, Max-Planck-Institut für Ornithologie, D-78315 Radolfzell, Germany; 4 Research Unit of Biodiversity (UO-CSIC-PA), Oviedo University, Campus of Mieres, 33600 Mieres, Spain; 5 School of Biological, Earth and Environmental Sciences, University College Cork, Cork, T12 YN60 Ireland

**Keywords:** Life history, Corticosterone, Tropical-temperate, Simulated territorial intrusion (STI), Seasonal

## Abstract

**Background:**

Latitudinal variation in avian life histories falls along a slow-fast pace of life continuum: tropical species produce small clutches, but have a high survival probability, while in temperate species the opposite pattern is found. This study investigated whether differential investment into reproduction and survival of tropical and temperate species is paralleled by differences in the secretion of the vertebrate hormone corticosterone (CORT). Depending on circulating concentrations, CORT can both act as a metabolic (low to medium levels) and a stress hormone (high levels) and, thereby, influence reproductive decisions. Baseline and stress-induced CORT was measured across sequential stages of the breeding season in males and females of closely related taxa of stonechats (*Saxicola* spp) from a wide distribution area. We compared stonechats from 13 sites, representing Canary Islands, European temperate and East African tropical areas. Stonechats are highly seasonal breeders at all these sites, but vary between tropical and temperate regions with regard to reproductive investment and presumably also survival.

**Results:**

In accordance with life-history theory, during parental stages, post-capture (baseline) CORT was overall lower in tropical than in temperate stonechats. However, during mating stages, tropical males had elevated post-capture (baseline) CORT concentrations, which did not differ from those of temperate males. Female and male mates of a pair showed correlated levels of post-capture CORT when sampled after simulated territorial intrusions. In contrast to the hypothesis that species with low reproduction and high annual survival should be more risk-sensitive, tropical stonechats had lower stress-induced CORT concentrations than temperate stonechats. We also found relatively high post-capture (baseline) and stress-induced CORT concentrations, in slow-paced Canary Islands stonechats.

**Conclusions:**

Our data support and refine the view that baseline CORT facilitates energetically demanding activities in males and females and reflects investment into reproduction. Low parental workload was associated with lower post-capture (baseline) CORT as expected for a slow pace of life in tropical species. On a finer resolution, however, this tropical-temperate contrast did not generally hold. Post-capture (baseline) CORT was higher during mating stages in particular in tropical males, possibly to support the energetic needs of mate-guarding. Counter to predictions based on life history theory, our data do not confirm the hypothesis that long-lived tropical populations have higher stress-induced CORT concentrations than short-lived temperate populations. Instead, in the predator-rich tropical environments of African stonechats, a dampened stress response during parental stages may increase survival probabilities of young. Overall our data further support an association between life history and baseline CORT, but challenge the role of stress-induced CORT as a mediator of tropical-temperate variation in life history.

**Electronic supplementary material:**

The online version of this article (doi:10.1186/s12862-017-0960-9) contains supplementary material, which is available to authorized users.

## Background

Environmental variation across latitudinal gradients has led to consistent differences in vertebrate life histories [1]. This is especially well studied in birds where tropical birds produce smaller clutches of offspring [2] and have lower basal metabolic rates [3, 4], but live longer [5, 6] than temperate birds. Such differences in pace of life between tropical and temperate birds [7] suggest a trade-off between current reproduction and longevity. Clutch size manipulation experiments in temperate birds have shown that reproduction in birds is indeed costly because enlarged broods can reduce future reproductive success and survival of parents (e.g. [8–12]). Costs of reproduction may arise because limited resources, for example energy, nutrients or time, cannot be allocated simultaneously to competing traits [13] and because of functional relationships between physiological systems [7, 14]. Therefore, it has been suggested that the evolution of life history traits is not only limited by environmental factors, but by physiological control systems that integrate environmental information [7]. In particular, endocrine control mechanisms may play an important role as mediators of life history trade-offs [7, 15–18].

The neuroendocrine stress axis represents a key interface between the external and internal environments of animals. Vertebrates respond to diverse challenges by activation of the hypothalamic-pituitary-adrenal (HPA) axis, which is highly conserved across vertebrate species. The HPA axis coordinates the physiological and behavioural responses to both energetically demanding situations and potentially life-threatening events [19–21]. The major effectors of the HPA axis are the glucocorticoids - the most important of which, in birds, is corticosterone (CORT, or cortisol in most mammals and fishes). CORT operates at distinct concentrations through binding to receptors of different affinity [19]. Baseline concentrations are thought to mediate the energetic needs of daily life and may be elevated during periods of high metabolic activity. In contrast, high concentrations occur when an individual is under stress and are thought to promote life-saving activities [21].

Baseline CORT concentrations are commonly upregulated during breeding (e.g. [22, 23], but see [24, 25] for examples where baseline CORT does not change during reproduction). Both experimental and correlational evidence suggests that elevated baseline CORT concentrations may prepare both sexes for increased energetic needs during the mating and parental phases [26–33]. Furthermore, parents with large broods and parents that feed their nestlings at high rates have higher baseline CORT concentrations than those with small broods and low feeding rates [28]. Thus, elevated baseline CORT concentrations during breeding can correlate with individual reproductive success [34–37]. On the other hand, elevated CORT levels after the breeding season have been shown to be negatively correlated with survival [38].

In response to unpredictable, life-threatening stressors, CORT concentrations can reach very high, “stress-induced” concentrations that may trigger an “emergency life history stage” [21]. This stage helps the individual to survive through physiological changes and by redirecting behaviour towards life-saving activities [21], although evidence for survival benefits is so far based on few studies [39, 40]. Support for this idea comes from observations that effects of stress-induced CORT are influenced by individual body condition. For example, experimentally increased high stress-induced CORT concentrations during periods of harsh weather can induce parents to leave their breeding sites temporarily or to abandon completely their current breeding attempt, especially if they have low body condition [41–43].

Thus, glucocorticoids are thought to mediate the trade-off between reproduction and survival by either promoting high reproductive output at the expense of future survival (elevated baseline concentrations) or by favouring immediate survival at the expense of current reproduction (high stress-induced concentrations). These effects of modulated levels of baseline and stress-induced CORT on reproductive success greatly depend on context, such as life history stage, prevailing environmental conditions, and a species’ life history (reviewed in [39, 40, 44]). For example, in female tree swallows *(Tachycineta bicolor)*, baseline CORT concentrations are negatively correlated with clutch mass and number of offspring when CORT is measured early during the incubation stage, but positively when CORT is measured during the nestling stage [34]. Similarly, the number of fledglings in house sparrows (*Passer domesticus*) were predicted by low pre-breeding, but high baseline CORT concentrations when feeding young [35]*.* The stress-induced CORT response can also be modulated during breeding, according to the value of a brood (“brood value hypothesis”, [42–44]). For example, house sparrows show a dampened CORT stress response when the value of their brood is experimentally increased [45], but an increased CORT stress response when the value of their brood is decreased [46]. An example for the importance of both, life history and environmental conditions, is provided by red crossbills (*Loxia curvirostra*), an opportunistic breeder that can breed both during summer and winter. In this species, individuals show a reduced stress response when in high breeding condition during winter, but not when breeding in summer [45]. Effects of CORT on adult survival may also depend on environmental context. For example, the stress response of non-breeding American redstarts *(Setophaga ruticilla)* predicts their return probability to their breeding sites when they overwinter in low quality scrub habitat, but not when they overwinter in high quality mangrove habitat [46].

Hence, despite general evidence that CORT mediates the trade-off between reproduction and survival, relationships between CORT and fitness are diverse, and a better understanding of the context that shapes them is required. There is clear need for clarifying studies that investigate CORT across a wide range of environments and life-history parameters. A particularly promising avenue for such research are the consistent differences in life histories between large-clutched, short-lived temperate and small-clutched, long-lived tropical birds [2, 5, 6]. Assuming that CORT mediates the underlying trade-offs, clear hypotheses for comparisons between temperate and tropical birds have been posited [17].

Short-lived, temperate species with a low probability of future breeding attempts should have higher baseline CORT concentrations, but lower stress-induced CORT concentrations than long-lived, tropical species, which have a higher probability of survival and that thus may have further breeding opportunities [17, 47]. In support of this hypothesis, in a comparative study between neotropical and North American temperate songbirds, Hau et al.[17] found that neotropical species indeed had lower baseline CORT concentrations than North American temperate birds and that stress-induced CORT concentrations positively co-varied with annual adult survival rate [17]. However, no detailed study so far has compared CORT concentrations of males and females of closely related species during their breeding activities at tropical and temperate latitudes [31].

Our study system, the common stonechat species complex *(Saxicola torquata),* has an extensive breeding range from Siberia to Southern Africa [48] and has played a major role in the development of the pace of life theory [7]. Stonechats show a robust latitudinal cline in metabolic rate, with genetically inherited, higher metabolic rates in higher-latitude populations [4, 49, 50]. Stonechats are socially monogamous seasonal breeders and defend a breeding territory regardless of latitude [48], but they also display a clear latitudinal cline in life history. Common garden experiments have shown that tropical stonechats have a genetically fixed smaller clutch size [51]. In the wild, tropical stonechats generally lay a single clutch, while temperate stonechats produce up to three consecutive clutches [48]. Tropical stonechats are residents or occasional altitudinal migrants and stay with the same partner year round, while migratory disposition of European stonechats varies from resident to migratory [48]. Local survival of tropical stonechats seems to be much higher than that of temperate stonechats: In previous studies the local apparent annual survival of stonechats in East Africa varied between 65–85% [52], while in a European population it was only 29–45% [53, 54]. Thus, stonechats are an ideal model system to study the endocrine mechanisms underlying differences in pace of life in a phylogenetically controlled setting [18].

Captivity studies under controlled conditions have strongly suggested associated clines in basal and stress-induced CORT and pace of life of different stonechat populations: fast-lived populations had higher baseline, and surprisingly, also higher stress-induced CORT than slow-lived populations [50]. However, it is unclear to which extent patterns would hold in actively breeding birds under natural conditions. Under field conditions, CORT patterns have been described for tropical stonechats [31, 55], but detailed data are in particular lacking for higher latitude populations. Furthermore, to address the context-dependence of CORT modulation, fine resolution information on sequential breeding stages is required [31]. Lastly, because earlier data were derived from very few populations, the generality of these patterns still needs to be tested.

To robustly test for hormonal differences with respect to pace of life, in this study we focused on the breeding season, when trade-offs occur, and collected comparative data from a large number of free-living stonechats. We compared baseline and stress-induced CORT concentrations of tropical stonechats from East Africa and temperate stonechats from Europe. We also included data from an island population from Fuerteventura (Canary Islands) that breeds at latitudes intermediate to mainland European and East African populations (although closer to mainland Europe than East Africa, [56, 57]) and that has a slow pace of life. Canary Islands stonechats *(Saxicola dacotiae)* are endemics of the semiarid island of Fuerteventura and have high local apparent annual survival (81%, [57]). Similar to East African stonechats, they breed during the rainy season, but may skip reproduction in very dry years. Clutch size (3–4 eggs) and the number of breeding attempts (0–2) depend on the amount of rain during the breeding season [56]. This present study is complementary to our recently published twin study on testosterone, another hormone implicated in life history trade-offs [18]. Samples used to determine the hormones in the two studies were obtained from the same populations and the same individual stonechats to a great extent.

Based on life history theory we hypothesized that CORT during breeding would differ between tropical, temperate and island stonechats according to their pace of life. Because tropical stonechats have a slower pace of life and are less fecund we predicted that they have lower baseline CORT concentrations than European stonechats. However, because of the short and highly synchronous breeding seasons in these tropical birds, male African stonechats could have elevated baseline CORT concentrations that are comparable to those of European males during the mating stages [18, 31].

With respect to breeding stage we expected similar within-species patterns in temperate and tropical stonechats. In females, we expected higher baseline CORT when incubating and feeding nestlings and fledglings than during mating in accordance with the potential role of baseline CORT as a mediator of parental investment. In males, we expected elevated CORT during the mating stages because of the short breeding seasons in both tropical and temperate stonechats [18, 31]. In addition, we expected higher baseline CORT when feeding young than during the incubation stage.

For stress-induced CORT, because the annual mortality of African stonechats is lower, we predicted that they should invest more into self-maintenance and be more risk sensitive and, therefore, have higher concentrations than European stonechats. Due to their slow pace of life, we expected that stonechats on Fuerteventura would have similar baseline and stress-induced CORT concentrations as East African stonechats.

## Methods

### Species and populations

Baseline and stress-induced CORT blood samples were collected from phylogenetically related stonechat taxa during their respective breeding seasons in tropical East Africa (*Saxicola torquata axillaris,* 8 populations, 112 males, 66 females, latitudes 0° - 4°S, altitudinal range: 1376–2500 m asl, sampled from 2012–2013; collectively referred to as African stonechats because of their breeding range within the tropics, i.e. within 23.5° of latitude), Europe (*Saxicola torquata rubicola*, 4 populations, 59 males, 30 females, latitudes 37° - 51°N, altitudinal range: 15–1350 m asl, sampled in 2013; collectively referred to as European stonechats), and Fuerteventura (*Saxicola dacotiae*, 13 males, 7 females, latitude 28°N, altitudinal range 57–359 m asl, sampled in 2013; referred to as Canary Islands stonechats). For detailed geographical locations and life history information see Additional file 1: Table S1. Stonechats from all East African populations lay a small clutch with on average three eggs, but breed at different times of the year corresponding to one of the two major rainy seasons. Southern populations breed from October to December [58], while Northern populations breed from March to July [59]. European stonechats (*Saxicola torquata*) breed from March to July [48], whereas Canary Islands stonechats breed according to rainfall from December/January to March [56].

We collected data on breeding biology, nestlings, and adults by observations and by capture. Breeding stage was categorized as pre-nesting, nest-building, egg-laying, incubation, nestlings and fledglings. Of these, combinations of pre-nesting, nest-building and egg-laying are collectively referred to as mating stage. Stages were determined by careful observation of pairs before capture (e.g. singing activity, nest-building activity by female, feeding activity of nestlings, presence of fledglings), and by assessing the presence or absence of a brood patch in captured females. For located nests, we recorded the number of eggs or nestlings and checked the nest again after a few days. We recorded geographical location and altitude using a GPS device. All individuals were measured (weight, tarsus and wing length), checked for moult, ringed with a numbered aluminium ring and a combination of three colour rings and were then released back into their territories. We determined the age of all individuals caught as either yearling (<2 years) or adult (≥2 years) based on feather moult pattern of the wings [60, 61].

### Capture methods

Stonechats were caught between 0700 h and 1800 h with baited clap net traps but also in some cases additionally lured by a mounted decoy and playback (simulated territorial intrusion (STI)). STIs have been previously used in stonechat studies [62–67]. Traps were observed continuously and upon capture birds were immediately removed from the traps. For STIs, one trap was fixed to the side of the decoy and several additional traps were baited and placed on the ground. According to an earlier study on African stonechats, the differences in trapping method affected CORT concentrations only for early breeding stages [31]. To further assess whether CORT is modulated in response to simulated territorial intrusions in stonechats, in Africa some individuals were caught using STI (time to catch (mean ± SD): 27 ± 22 min, Table 1), while others were caught with baited clap net traps only (time to catch (mean ± SD): 46 ± 36 min, Table 1). In Europe, all males were caught during simulated territorial intrusions (time to catch (mean ± SD): 36 ± 30 min, Table 1). To elicit a territorial response a remote-controlled loudspeaker (Foxpro Scorpion, digital game caller, FOXPRO Inc. Lewistown, USA) was put underneath a decoy to play back the territorial song of a potential rival at a sound pressure level of 65 dB SPL at 1 m (as measured with a CEL 573.B1 Sound Level Analyser). In Europe we used playbacks downloaded from the British library or from http://www.xeno-canto.org/. In Africa, we used playback songs, which were recorded from the same population. All females were caught with baited clap net traps (time to catch (mean ± SD): Africa: 38 ± 30 min, Europe: 54 ± 39 min, Table 1), but were in some cases caught during a catching event of their mates and, hence, sometimes exposed to STIs before capture. Male and female Canary Islands stonechats were caught with baited clap net traps without playback (time to catch (mean ± SD): 21 ± 23 min, Table 1). For a summary of the different capture methods that were used in the different breeding regions and in the two sexes see Table 1.

**Table 1:** Overview of sample sizes by sex, capture method, and breeding stage during which individuals were caught in the different breeding regions (Africa, Europe, Canary Islands)

Region, sex	Capture method	Mating			Incubation	Young	
		Pre-nesting	Egg-laying	Nest-building		Nestlings	Fledglings
Africa, males	baited clap net traps	0	0	3	10	12	15
	STI	18	4	9	16	11	14
Africa, females	baited clap net traps	1	1	6	23	18	17
Europe, males	STI	NA	9	2	31	4	10
Europe, females	baited clap net traps	NA	10	3	7	6	4
Canary Islands, males	baited clap net traps	NA	NA	NA	7	1	5
Canary Islands, females	baited clap net traps	NA	NA	NA	3	1	3

NA indicates non-available data

### Plasma separation and hormone analysis

To measure baseline concentrations of CORT an initial blood sample (~120 μl) was taken within 3 min of capture (mean ± SD: Africa males: 120 ± 32 s, Africa females: 124 ± 36 s; Europe males: 119 ± 40 s, Europe females: 111 ± 33 s, Canary Islands males: 105 ± 34 s, Canary Islands females: 107 ± 35 s; [68]) by venipuncture of the wing vein or (less often) with a syringe from the jugular vein and collected into heparinized capillaries. CORT concentrations sampled from the wing or jugular vein have been shown to be similar in male song sparrows *(Melospiza melodia)* [69]. To measure stress-induced CORT, individuals were kept in an opaque cloth bag and a second blood sample (~50 μl) was taken 30 min after capture. In both instances, plasma was immediately separated by centrifugation with a Compur Minicentrifuge (Bayer Diagnostics) or a Spectrafuge Mini Laboratory Centrifuge (Labnet International, Inc.). The amount of plasma was measured with a Hamilton syringe and stored in 500 μl pure ethanol [70]. After the end of each field season (≤4 months) samples were stored at −80°C. CORT concentrations were quantified using an enzyme immunoassay kit (EIA, Cayman #500655) according to the manufacturer’s instructions. The kit has been previously validated in another songbird, the dark-eyed junco (*Junco hyemalis*, [71]) and we further established parallelism between a standard curve based upon reference standards provided by the kit and stonechat plasma dilution curves (x1, x2, x4, x8, x16) using stress-induced samples from two individuals. Briefly, 1000 μl dH_2_0 and 10 μl of tritiated CORT (~8000 cpm) were added to each sample, and then samples were allowed to equilibrate overnight. Samples were extracted twice with 4 and 2 ml of diethyl ether, respectively, dried with N_2_ and reconstituted with 300 μl assay buffer. Baseline samples were diluted 1:4 and stress-induced samples 1:10 prior to plating in duplicate. A pool of zebra finch plasma was extracted together with the samples and included in each plate undiluted and in a 1:4 dilution to assess intra- and inter-assay variation. The concentration of CORT in plasma samples was calculated by using a standard curve run in duplicate on each plate and corrected for incomplete recoveries (average extraction efficiency: 71 ± 7%). Samples were quantified in 18 assays, each containing samples from African and European populations with baseline and stress-induced samples from the same individual on the same plate. Inter-assay variation ranged from 13.6% (undiluted internal control, *n* = 18) to 17.9% (1:4 diluted internal control, *n* = 18) and intra-assay variation ranged from 1.6 to 17.5% (average: 10.1%, *n* = 18).

### Statistical analysis

Data were analysed within the R environment (R version 3.2.2, [72]) and the packages arm [73], JAGS [74] and runjags [75]. Bayesian linear mixed models (see below for more information) were used to determine whether variation in CORT was related to breeding region (tropical Africa, temperate Europe, Canary Islands), breeding stage or the interaction between breeding region and breeding stage. Population identity within a breeding region was included as a random intercept.

Baseline and stress-induced CORT concentrations were natural log-transformed prior to analysis and predictor covariates (scaled mass index, time of day, playback duration, handling time) were centred to a mean of zero. Handling time refers to the time elapsed between capture and the initial baseline blood sample. As handling time was similar for all stress-induced samples (~30 min after capture) it was not included in the analysis of stress-induced CORT concentrations. Predictor covariates and their interactions were removed from the models when they did not explain a significant amount of variation in the data (i.e. credible intervals included zero). Predictor variables that were part of the experimental set-up / hypothesis (breeding region, breeding stage) were always retained in the models. CORT data were analysed over several subsets. Data of males and females were analysed separately because of their different reproductive roles and because capture methods differed (males: STI or baited traps; females: baited traps, Table 1). In males, we checked for effects of capture method and breeding stage, using African stonechats which had the greatest range of capture methods and breeding stages (Table 1). We then compared males of African and European populations, and African and Canary Island stonechats, respectively, using subsets with matched capture methods and breeding stages (Table 1). In females, we first compared African and European populations, which had the greatest range of breeding stages, and then we compared Canary Island stonechats to both populations (Table 1). Finally, we also analysed the relationship of CORT between mates that were sampled under comparable conditions, i.e. during the same catching event.

### CORT males

Baseline CORT concentrations were analysed for differences between capture method (STI, control) and breeding stage in African males. Breeding stage was included as a factor with four levels: mating (comprising pre-nesting, nest-building and egg-laying), incubation, nestlings and fledglings (Table 1). Because of low sample sizes during the mating stage in control birds (*n* = 3) we did not include a capture method*breeding stage interaction in the model. In a next step, we analysed differences in CORT concentrations between African and European males. Because capture method had a significant influence on CORT in African males, and European males were only caught using STIs (Table 1), only African males that had been caught with STI were included in the comparison between African and European males. In this comparison, breeding stage was included as a factor with three levels: mating (nest-building and egg-laying), incubation and young (nestlings and fledglings, Table 1). In Europe, no males had been caught during pre-nesting (Table 1) and we, therefore, restricted the mating stage to nest-building and egg-laying, resulting in smaller sample sizes for African males compared to the Africa only analysis. Effects of breeding region, breeding stage and any interaction between breeding region and breeding stage were investigated for baseline and stress-induced CORT. Further, we tested whether stress-induced CORT was dependent on baseline CORT and whether these relationships differed between breeding regions. Finally, males of the Canary Islands stonechat and males from Africa that were caught without STI were compared using linear models (no random intercept). European males were not included as they had been caught exclusively with STIs. Samples were restricted to incubation and feeding of young stages, and breeding stage was not included as a factor (Table 1). Because of the positive effect of STIs on CORT concentrations in males, we refer to CORT concentrations directly after capture as post-capture CORT instead of baseline CORT concentrations.

### CORT females

Baseline and stress-induced CORT concentrations were tested for differences between European and African females. We included in the model the factors breeding region, breeding stage, and the interaction between breeding region and breeding stage. Breeding stage was defined as a factor with the three levels mating (including nest-building and egg-laying), incubation and young (including nestling and fledgling stages, Table 1). Females of the Canary Islands stonechat were included in linear models (no random intercept) that were restricted to the incubation and feeding of young stages. Breeding stage was not included as a factor because of small sample sizes for Canary Islands stonechats for the different breeding stages (Table 1). In a last step, CORT concentrations of females were tested for correlations with CORT concentrations of their male mates using linear models. In these models, data from all populations were used. The analysis was restricted to pairs that were caught during a single catching event which in some cases involved STI (*n* = 61).

Body condition for African, European and Canary Islands stonechats was estimated separately for males and females using each individual’s body mass and tarsus length in a scaled mass index (SMI, following Peig and Green (2010) [76]). The SMI analysis standardizes body mass at a fixed value of a linear body measurement based on the scaling relationship between mass and length, in our case tarsus length. This value is derived from empirical values, in our case on data from 627 individuals (Africa: males *n* = 122, females *n* = 85, Europe: males *n* = 193, females *n* = 108, Canary Islands: males *n* = 82, females *n* = 37) including individuals for which we had not collected CORT samples. The scaling exponent (b_sma_) was estimated through standard major axis regression of body mass against tarsus length on a natural log scale. We determined b_sma_ and mean tarsus length required for the calculation of the SMI for each sex and breeding region separately. In European and Canary Islands stonechats the b_sma_ and mean tarsus length calculations included also data from long-term ringing data sets.

We chose a Bayesian approach to draw inferences from the models because frequentist methods do not allow calculating accurate degrees of freedom in mixed model analysis [77]. Bayesian statistics estimate probability distributions of the parameters in the model (i.e. posterior distributions) given the data and prior knowledge about the distribution of the data (specified as priors) [78]. Model parameters were estimated as the mean of their posterior distributions, and the 2.5% and 97.5% credible intervals. Minimally informative priors for both mean (dnorm (0, 10^^−6^)) and variance (dgamma (0.001, 0.001)) parameters were used, i.e. we assumed no prior knowledge about the factors in our models. Marcov-Chain Monte-Carlo (MCMC) simulations were checked for convergence of chains using trace plots and psrf values [79]. Effective sample sizes were > 15000 in all cases. Model residuals were graphically checked for violations of model assumptions (normality, heteroscedasticity, autocorrelations) [78]. Data are presented as back-transformed means and their 95% Bayesian credible intervals in figures and as the difference and 95% Bayesian credible interval (in squared brackets) from the mean intercept (on a natural log scale) in tables. Bayesian statistics do not produce test statistics or *p*-values, however, when the Bayesian 95% credible interval of the difference between two means does not include zero, this can be interpreted as a detectable difference and the real estimate lies with a probability of 95% within the Bayesian credible interval [80]. Similarly, when the 95% Bayesian credible interval of the slope in a regression does not include zero a meaningful relationship between the continuous predictor and the dependent variable can be assumed [78].

## Results

### Males: effects of capture method and breeding stage on CORT of African males

**Fig. 1:**
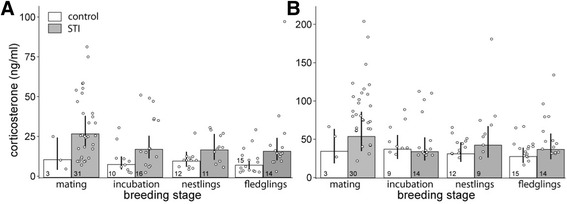
Effects of capture method on (**a**) post-capture and (**b**) stress-induced CORT concentrations of African male stonechats caught during different breeding stages. Males caught in a control situation with a baited trap had lower CORT concentrations than males caught during simulated territorial intrusions (STI) with playback and decoy. Although control and STI birds are presented separately during each breeding stage, we had not included a capture method*breeding stage interaction in the model because of low sample sizes for control birds during mating. Bars and error bars represent back-transformed posterior means and their 95% Bayesian credible intervals, open dots represent data points from individuals. Different populations were included as random intercepts in the models. Sample sizes are given inside bars

Male African stonechats that were confronted with a STI had overall higher post-capture and stress-induced CORT concentrations than males that were caught with baited traps (Table 2, Fig. 1). Considering all males, i.e. control and STI birds combined, post-capture and stress-induced CORT concentrations were higher during mating than during incubation and when feeding fledglings (Table 2, Fig. 1). During the nestling stage, CORT tended to be lower than during mating, but the credible interval of the difference between the mating and nestling stage for post-capture CORT crossed zero (Table 2). Scaled mass index, age and time of day were not correlated with post-capture and stress-induced CORT concentrations nor did handling time influence post-capture CORT concentrations (Table 2).

**Table 2:** CORT concentrations of male African stonechats in relation to capture method (control, STI) and breeding stage

	Post-capture CORT	Stress-induced CORT
	Estimates and 95% credible intervals	Estimates and 95% credible intervals
Intercept: Control, mating stage	2.6 [2.2, 3.0]	4.1 [3.8, 4.3]
Capture method (STI, control)	**0.7 [0.4, 1.0]**	**0.3 [0.1, 0.5]**
Incubation	**−0.5 [−0.9, −0.04]**	**−0.4 [−0.6, −0.1]**
Nestlings	−0.4 [−0.8, 0.06]	**−0.3 [−0.6, −0.05]**
Fledglings	**−0.5 [−0.9, −0.08]**	**−0.4 [−0.7, −0.2]**
Scaled mass index	−0.05 [−0.2, 0.07]	−0.05 [−0.1, 0.03]
Age yearling	−0.2 [−0.6, 0.1]	−0.1 [−0.3, 0.1]
Time of day	0.0002 [−0.0003, 0.0007]	−0.0002 [−0.0005, 0.0002]
Handling time	0.003 [−0.001, 0.008]	

Data shown are estimates and 95% Bayesian credible intervals of natural log-transformed CORT concentrations. Estimates are relative to the intercept as reference level, which here is the mating stage of controls. The left column shows the post-capture and the right column stress-induced CORT, each with the corresponding Bayesian mean estimate and its’ credible intervals. Estimates of the cofactors capture method and breeding stages refer to differences from the intercept estimate. When 0 (zero) is not included in the credible intervals there is a detectable effect of this parameter on the dependent variable (shown in bold)

### Males: comparison of CORT and body mass index between African and European stonechats

**Fig. 2:**
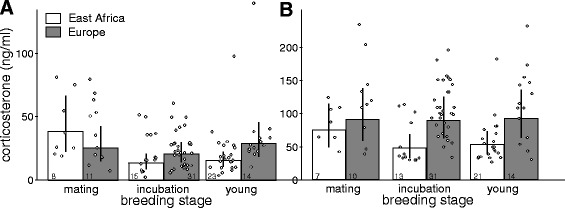
Effects of breeding region and breeding stage on (**a**) post-capture and (**b**) stress-induced CORT concentrations of males. Data show CORT concentrations of African and European male stonechats caught with STI. Bars and error bars represent back-transformed posterior means and their 95% Bayesian credible intervals

African and European males, which all were caught by STI, did not differ overall in their post-capture CORT concentrations, but instead, showed differences relating to breeding stage. Post-capture CORT concentrations of African males were higher during mating than during incubation or when feeding young (Table 3, Fig. 1 and 2a). In contrast, post-capture CORT concentrations of European males did not decrease with breeding stage. Consequently, European males had higher post-capture CORT concentrations than African males when feeding young (Europe*young, Table 3, Fig. 2a). Time between start of playback and capture (STI duration) was positively correlated with post-capture CORT concentrations: birds that had been exposed to playback longest had the highest post-capture CORT concentrations when captured (Table 3). Scaled mass index, age, time of day and handling time had no detectable influence on post-capture CORT concentrations (Table 3). European males had higher stress-induced CORT concentrations than African males (Table 3, Fig. 2b). Breeding stage, the interaction between breeding stage and breeding region, scaled mass index, age and time of day had no detectable influence on stress-induced CORT concentrations (Table 3, Fig. 2b).

**Table 3:** CORT concentrations of male stonechats in relation to breeding region and breeding stage

	Post-capture CORT	Stress-induced CORT
	Estimates and 95% credible intervals	Estimates and 95% credible intervals
Intercept: Africa, mating stage	3.7 [3.2, 4.2]	4.2 [3.8, 4.6],
Europe	−0.6 [−1.2, 0.1]	**0.5 [0.08, 0.9]**
Incubation	**−0.8 [−1.4, −0.3]**	−0.2 [−0.4, 0.1]
Young	**−0.8 [−1.2, −0.3]**	−0.1 [−0.4, 0.2]
Europe*incubation	0.5 [−0.2, 1.3]	0.4 [−0.2, 1.0]
Europe*young	**1.0 [0.2, 1.7]**	0.4 [−0.2, 1.0]
Scaled mass index	−0.09 [−0.2, 0.04]	−0.03 [−0.1, 0.07]
Age yearling	−0.07 [−0.5, 0.3]	−0.1[−0.4, 0.2]
STI duration	**0.007 [0.002, 0.01]**	
Time of day	0.0002 [−0.0002, 0.0007]	0.0001 [−0.0002, 0.0004]
Handling time	0.002 [−0.002, 0.006]	

Data shown are estimates and 95% Bayesian credible intervals of natural log-transformed CORT concentrations. Estimates are relative to the intercept as reference level, which here is the mating stage of African males. The left column shows the post-capture and the right column stress-induced CORT, each with the corresponding Bayesian estimate and its’ credible intervals. Estimates of cofactors refer to differences from the intercept estimate. When 0 (zero) is not included in the credible intervals there is an effect of this parameter on the dependent variable (shown in bold). In the case of a significant interaction, the estimated difference of the interaction term is added to the difference of the main effect from the intercept

**Fig. 3:**
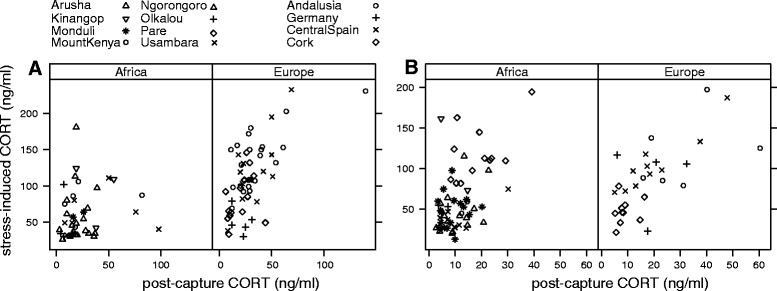
Relationship between post-capture and stress-induced CORT concentrations of (**a**) male and (**b**) female stonechats separated by breeding region. Stress-induced CORT concentrations correlated positively with post-capture CORT concentrations in European males and females and in African females, but not in African males. All males were caught with STI. Females were caught with baited traps. Symbols represent data points from individuals from different populations

Stress-induced CORT concentrations correlated positively with post-capture CORT concentrations in European, but not in African male stonechats (Africa (intercept): 3.8 [3.1, 4.4], slope all: 0.1 [−0.06, 0.3], slope Europe: 0.3 [0.07, 0.6], Fig. 3a).

Scaled mass index of African males was higher than that of European males (Africa (intercept): 16.3 [15.9, 16.7], difference Europe: −1.8 [−2.3, −1.4]). Scaled mass index of all males was lower during the nestling stage than during mating (mating (intercept): 16.3 [15.9, 16.7], difference nestlings: −0.5 [−0.8, −0.3]).

### Males: comparison of CORT between African and Canary Islands stonechats

Considering only males caught during incubation and feeding of young and without STI, post-capture and stress-induced CORT concentrations of Canary Islands males were higher than those of African males (post-capture: Africa (intercept): 2.1 [1.9, 2.3], difference Canary Islands: 1.1 [0.7, 1.5], stress-induced: Africa (intercept): 3.7 [3.6, 3.9], difference Canary Islands: 0.7 [0.4, 1.0], Table 4). Scaled mass index did not correlate with CORT (post-capture: slope: −0.1 [−0.3, 0.1], stress-induced: slope: −0.1 [−0.2, 0.1]). Handling time was positively correlated with post-capture CORT (0.006 [0.0003, 0.01]).

**Table 4:** Comparison of CORT concentrations of Canary Islands stonechats with those of African and European stonechats

	Africa	Canary Islands	Europe
Males	ng/ml	ng/ml	ng/ml
Post-capture CORT	7.9 [6.4, 9.8]	23.1 [12.4, 42.9]	NA
Stress-induced CORT	42.1 [35.9, 49.1]	84.6 [53.8, 132.6]	NA
Females			
Post-capture CORT	9.5 [7.9, 11.4]	16.6 [8.0, 34.1]	13.7 [7.8, 23.9]
Stress-induced CORT	51.0 [43.4, 60.1]	107.2 [57.3, 200.5]	60.8 [37.5, 98.5]

Data show back-transformed CORT concentrations (mean and 95% Bayesian credible intervals) of male and female African, Canary Islands and European stonechats during incubation and when feeding young. All individuals were caught without STI. As European males were always caught with STI, they were not included in the comparison with Canary Islands stonechats

### Females: comparison of CORT and body mass index between African and European stonechats

**Fig. 4:**
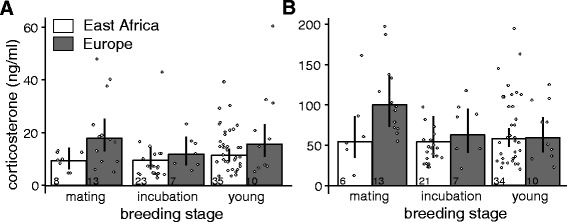
Effects of breeding region and breeding stage on (**a**) post-capture and (**b**) stress-induced CORT concentrations in females. Data show CORT concentrations of African and European female stonechats caught during different breeding stages. European females had overall higher post-capture and stress-induced CORT concentrations than African females. Although African and European birds are presented separately during each breeding stage, an effect of breeding stage or a breeding region*breeding stage interaction were not detectable. Bars and error bars represent back-transformed posterior means and their 95% Bayesian credible intervals

European females had higher post-capture CORT concentrations than African females (Table 5, Fig. 4a). Post-capture CORT concentrations did not differ with breeding stage and no interaction between breeding region and breeding stage was found (Table 5, Fig.4a). Although females were bled within 3 min, handling time was positively correlated with post-capture CORT (Table 5) and removal of two females that had been bled outside 3 min did not change the effect. Scaled mass index, age, time of day and exposure to STI had no detectable influence on post-capture CORT concentrations (Table 5). European females had slightly higher stress-induced CORT concentrations than African females (Table 5, Fig. 4b). Breeding stage and the interaction between breeding region and breeding stage had no detectable influence on stress-induced CORT concentrations (Table 5, Fig. 4b). Time of day was positively correlated with stress-induced CORT. Scaled mass index and age had no detectable influence on stress-induced CORT concentrations (Table 5).

**Table 5:** CORT concentrations of female stonechats in relation to breeding region and breeding stage

	Post-capture CORT	Stress-induced CORT
	Estimates and 95% credible intervals	Estimates and 95% credible intervals
Intercept: Africa, mating stage	2.2 [1.8, 2.6]	3.9 [3.4, 4.3]
Europe	**0.6 [0.08, 1.2]**	**0.7 [0.008, 1.4]**
Incubation	−0.3 [−0.6, - 0.01]	0.04 [−0.4, 0.5]
Young	0.2 [−0.2, 0.5]	0.2 [−0.2, 0.6]
Europe*incubation	0.005 [−0.6, 0.6]	−0.4 [−1.0, 0.2]
Europe*young	0.03 [−0.6, 0.7]	−0.5 [−1.1, 0.1]
Scaled mass index	−0.03 [−0.1, 0.02]	−0.01 [−0.06, 0.04]
Age yearling	0.04 [−0.2, 0.3]	0.07 [−0.1, 0.3]
Time of day	−0.00002 [−0.0004, 0.0003]	**0.0005 [0.0002, 0.0008]**
Handling time	**0.007 [0.004, 0.01]**	
Exposure STI	0.1 [−0.2, 0.4]	

Data shown are estimates and 95% Bayesian credible intervals of natural log-transformed CORT concentrations. Estimates are relative to the intercept as reference level, which here is the mating stage of African females. The left column shows the post-capture and the right column stress-induced CORT, each with the corresponding Bayesian estimate and its’ credible intervals. Estimates of cofactors refer to differences from the intercept estimate, which here is the mating stage of African females. When 0 (zero) is not included in the credible intervals there is an effect of this parameter on the dependent variable (shown in bold)

Stress-induced CORT concentrations correlated positively with baseline CORT concentrations and this relationship did not differ between regions (Africa (intercept): 3.2 [2.7, 3.7], slope all: 0.4 [0.3, 0.6], interaction: 0.04 [−0.3, 0.4], Fig. 3b).

African females had higher scaled mass indices than European females (Africa (intercept): 17.9 [17.2, 18.6], difference Europe: −0.8 [−1.5, −0.1]). All females had higher scaled mass indices during mating (nest-building and egg-laying) than during all other stages (mating (intercept): 17.9 [17.2, 18.6], difference incubation: −0.9 [−1.8, −0.05], difference nestlings: −2.1 [−2.8, −1.4], difference fledglings: −1.8 [−2.7, −0.8]).

### Females: comparison of CORT between African, European and Canary Islands stonechats

Post-capture CORT concentrations of females caught during the parental stages (incubation, nestlings, and fledglings) were higher in European and Canary Islands than in African females (Africa (intercept): 2.2 [2.1, 2.4]), difference Europe (0.4 [0.03, 0.7]), difference Canary Islands (0.6 [0.05, 1.1], Table 4). Canary Islands females had higher stress-induced CORT concentrations than African or European females (Africa (intercept): 3.9 [3.8, 4.1], difference Europe: 0.2 [−0.1, 0.5], difference Canary Islands: 0.7 [0.3, 1.2], Table 4). Handling time was positively correlated with post-capture CORT (0.006 [0.0008, 0.01]). Scaled mass index was not correlated with post-capture or stress-induced CORT (post-capture: slope: −0.05 [−0.1, 0.04], stress-induced: slope: 0.001 [−0.08, 0.09]).

### Males and females: correlations between mates

**Fig. 5:**
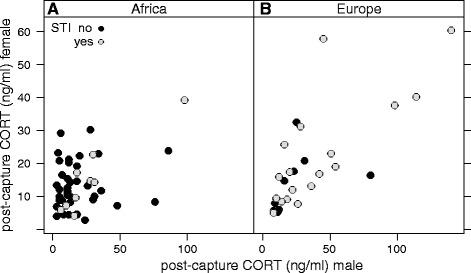
Correlations between post-capture CORT concentrations of females and their male partners in (**a**) African and (**b**) European stonechats. Female post-capture CORT concentrations correlated positively with post-capture CORT concentrations of their mates but only when both mates were exposed to STI

Post-capture CORT concentrations of females correlated strongly and positively with post-capture CORT concentrations of their mates, but only when both were caught during the same catching event that involved a simulated territorial intrusion into the pair’s territory (intercept: 2.2 [1.7, 2.7], slope (CORT male): 0.1 [−0.07, 0.3], slope (Cort male*STI): 0.5 [0.2, 0.9], Fig. 5).

## Discussion

Overall our findings on CORT of male and female stonechats at tropical and temperate sites only partly support predictions based on differences in pace of life. European stonechats have a faster pace of life than African and Canary Islands stonechats. In line with their faster pace of life, higher reproductive investment and parental workload, European females during all breeding stages and European males during parenting had higher post-capture (baseline) CORT concentrations than African females and males. However, during nest-building and egg-laying post-capture CORT concentrations of African males were similar to those of European males. Furthermore, despite their lower annual reproductive investment and mortality rate, African stonechats had lower stress-induced CORT concentrations than European stonechats. Likewise, we found relatively high post-capture and stress-induced CORT concentrations in slow-paced Canary Islands stonechats. Below we discuss these findings in detail for the contexts of parental workload, territory defence and breeding-stage specific stress responses.

### Baseline CORT and parental workload

Breeding is an energetically demanding life-cycle stage. In female songbirds, basal metabolic rate and energy expenditure are elevated both during egg-laying and when feeding nestlings, and both are also dependent on brood size [81, 82]. Baseline CORT is thought to regulate energetic needs for daily activities [83] and consequently, baseline CORT is upregulated during breeding in many species [22]. This is further supported by studies, in which experimentally elevated CORT increased foraging effort and nestling provisioning rates [27, 29]. Additional support is provided by a brood size manipulation study, in which females of enlarged broods fledged more young and had higher baseline CORT than females of control broods [33]. Furthermore, recent studies in female house wrens *(Troglodytes aedon)* and European starlings *(Sturnus vulgaris)* suggest that CORT could also influence fecundity (clutch size and number of clutches) [26, 84]. The facilitation of fecundity and parental investment by baseline CORT is supported by the results of the current study where post-capture CORT was found to be higher in European stonechats, which lay more eggs per clutch than African stonechats. All breeding stages combined, African females had lower post-capture CORT than European females. In European males, this contrast was found only when the birds were feeding young. Canary Islands stonechats also had higher post-capture CORT concentrations than African stonechats, although they generally share a slow pace of life. Canary Islands stonechats lay larger clutches during wet (3.7 eggs) than during dry years (2.7 eggs) and are sometimes double-brooded in favourable seasons [56]. For this study, Canary Islands stonechats were caught in a year with good rainfall (2013), which may have resulted in clutches that were larger than those of African stonechats (3.0 eggs). Our results, thus, suggest that the higher energetic costs of laying larger clutches in females and of feeding more nestlings and fledglings in both parents are likely related to higher post-capture CORT concentrations. Similarly, a study on temperate blue tits *(Cyanistes caeruleus)* found that larger-clutched blue tits breeding on mainland Southern France had higher baseline CORT than small-clutched blue tits breeding on the Island of Corsica [85].

An increased parental work load is commonly associated with physiological costs for birds. For example, increased energy expenditure can have a negative impact on parental fitness through increased production of reactive oxygen species (ROS). ROS can lead to oxidative stress – i.e. oxidative damage to DNA, lipids and proteins [86]. Further, increased parental workload can lead to higher levels of blood parasites and reduced resistance to oxidative stress [87]. Thus, high parental workload may ultimately affect parental survival and should hence be kept low when low mortality allows repeated breeding, and when the value of clutches is low. Our findings of low post-capture CORT in African stonechats supports the general idea of low baseline CORT associated with low reproductive investment. In African stonechats, a long lifespan allows for multiple breeding years, but in each year, the value of a brood may be low because predation on young is high [7, 55]. We speculate that these life history aspects have selected for overall low baseline CORT levels during the breeding season.

Common garden experiments in stonechats have also shown that tropical stonechats have lower CORT and resting metabolic rates than stonechats from the Northern hemisphere, and that this latter difference is heritable but sensitive to life-history stage [4, 49, 50, 88, 89]. One study investigated direct relationships between metabolic rates and CORT and found support for associations on the level of population, but not of individuals [50], thus overall supporting the idea of an involvement of CORT in differences of pace of life.

### Post-capture CORT and simulated territorial intrusions

An additional axis of variation in post-capture CORT levels was associated with territorial defense. African males had higher post-capture CORT concentrations when caught after simulated territorial intrusions than when caught with food-baited traps. This confirms similar findings from an earlier study on African male stonechats [31]. In this earlier study, STI was associated with elevated male post-capture CORT concentrations only during the pre-breeding stage. In our study, the effect of STI also appeared to be greatest during early breeding stages, but we did not detect a significant interaction between breeding stage and capture method. CORT has been shown to increase during simulated territorial intrusions in other songbirds [90–94]. This elevation in CORT following STIs is perhaps not surprising as CORT mediates processes that provide the body with readily available energy during challenging situations [83]. Further, CORT can increase context-dependent activity levels [95]. Thus, the high CORT concentrations during STIs reported in the two studies on African stonechats may facilitate a highly active state and the increased energetic needs of defending a territory [31].

Both tropical and temperate stonechats breed seasonally. Species with short breeding seasons breed more synchronously than species with extended breeding seasons, and consequently may face a higher risk of extra-pair matings [96]. In particular African males had higher CORT concentrations during mating than during other breeding stages. This peak in CORT concentrations coincides with a peak in testosterone concentrations identified in our twin study on overlapping populations and in other sources. Testosterone peaked during nest-building [18, 31, 66] and the time when male stonechats are most aggressive [18]. In contrast, for European stonechats we could not confirm a similar, mating-related, peak in post-capture CORT, although seasonal testosterone profiles did not differ between males from different latitudes according to our twin study [18]. Generally, the beginning of the breeding season is a highly demanding and potentially stressful time for birds. Fitness gains by preventing extra-pair copulations of their mates may outweigh the costs of elevated baseline CORT levels for males during this short period of time and similar demands may also affect females. Earlier studies on European stonechats have shown that females are highly responsive to song replay, and that pair interactions may raise CORT concentrations of females in response to territorial intrusions [65, 97]. Our CORT data from paired stonechats support the idea that territorial intrusions affect both mates, as post-capture CORT concentrations of females that were caught during simulated territorial intrusions of their male partners were highly correlated with those of their mates.

### Stress-induced CORT, survival and brood value

In addition to its role as a metabolic hormone, CORT is one of the major mediators of the stress response in vertebrates. Within minutes of the perception of a stressor, circulating CORT concentrations rise dramatically [98] and facilitate behaviours that promote self-maintenance and survival [21, 42]. Evidence suggests that stress-induced CORT concentrations are modulated with respect to risk to self, as well as with respect to the value of a brood [47, 99]. Because tropical species are longer-lived and have a lower reproductive investment than temperate species, it has been proposed that tropical species should be more risk-sensitive and hence their stress response should be higher than that of temperate species [7]. However, although adult mortality is lower in African than in European stonechats [52, 54], in the current study, African stonechats had lower stress-induced CORT concentrations than European stonechats. These findings correspond with common garden studies of four of the stonechat populations studied here, which also found that stress-induced CORT concentrations were lowest in African stonechats [50]. A possible reason is that although fecundity (clutch size and number of broods) is usually higher in temperate environments, investment into individual offspring – both during the nestling stage and post-fledging – may be higher in tropical environments leading to higher quality offspring in tropical birds [100, 101]. For example, African stonechats take care of their fledglings for at least six weeks, while European stonechats overlap clutches and initiate a second clutch when fledglings are only two weeks old [55, 102]. Field studies suggest that predators are a main reason for this extended parental care and possibly for single-broodedness in African stonechats [55, 103], where especially males actively guard their fledglings against predators. A comparative study showed that males in territories where shrikes – the main predators of fledglings - were present had a lower body condition and elevated baseline CORT compared to males in territories without shrikes [55]. High stress-induced CORT can induce parents to leave their brood [104]. Because of their relatively short breeding season, tropical stonechats may not be able to re-nest when their brood fails during the fledgling stage. Thus, in our tropical region, where predators on fledglings are plenty, the prolonged parental care provided by stonechats may be critical for fledged young to reach independence, and hence, be facilitated by a dampened CORT stress response. Supporting this idea further, stress-induced CORT concentrations of male African stonechats decreased with breeding stage and were lower when they had fledglings than during mating. A dampened stress-response during the nestling/fledgling stage may be a more general pattern in challenging environments where re-nesting possibilities are scarce. For example, in Arctic-breeding sparrows males had a lower CORT response to handling stress when feeding nestlings than before eggs hatched [105]. In Arctic regions where storms and harsh weather occur frequently during the breeding season, a dampened stress response may prevent parents from too readily jeopardizing their brood without possibilities to re-nest [106].

In accordance with their high annual survival, male Canary Islands stonechats had higher stress-induced CORT than African and European stonechats. As common for many island species, nest-failure due to predation on young is lower in Canary Islands than in African and European stonechats. Nest failure was especially low before the introduction of predatory mammals [107, 108], which now are the main causes of nest failure in the Canary Islands stonechat (29% of nests failed; 67% of these predation events were due to feral cats during 2000–2003, [56, 109]). Thus, introduced predatory species may exert a selective pressure that may dampen the CORT stress-response in the future. Together with captive studies from stonechats [50] and swamp sparrow subspecies *(Melospiza sp.)* [110] our data suggest that the CORT stress response has a genetic basis and responds to selection pressures associated with environmental conditions and alternative life histories.

## Conclusions

Our data support the view that baseline CORT concentrations facilitate energetically demanding activities in males and females and reflect investment into reproduction. Reduced parental workload because of small clutches and consequently lower concentrations of circulating CORT may, thus, contribute to a slow pace of life in tropical species. However, our data call for a refinement of this classical conclusion of tropical-temperate comparisons: the patterns can differ or even be reversed during specific life-cycle stages. In species with short breeding seasons a potentially high risk of extra-pair copulations may favour high baseline CORT concentrations at the beginning of the breeding season in particular in tropical males. Likewise, contrasting with the hypothesis that species with high annual survival and a slow pace of life should be more risk-sensitive, tropical stonechats had lower stress-induced CORT concentrations than temperate stonechats during the breeding season. A dampened stress response during parental stages may increase survival probabilities of young in predator-rich tropical environments. Overall, our data further support a mediating role of baseline CORT relative to reproductive effort. In contrast, stress - induced CORT did not follow the predictions from life history theory, nor classical tropical-temperate patterns. To resolve these uncertainties, more studies are needed that reveal which environmental factors have shaped the CORT stress response and its consequences for avian fitness.

## Additional files

Additional file 1: Table S1. Geographical and life history information of populations studied. (DOCX 18 kb)

Additional file 2: Datasets analyzed during the current study. (XLSX 113 kb)

## Abbreviations

SMI: Scaled mass index
STI: Simulated territorial intrusion
