# Supplementary material for: Baseline and stress-induced levels of corticosterone in male and female Afrotropical and European temperate stonechats during breeding
Source: BMC Evol Biol. 2017 May 22;17:114. doi: 10.1186/s12862-017-0960-9 (PMC5441054; doi:10.1186/s12862-017-0960-9)
Supplement: Supplementary file 1 — Geographical and life history information of populations studied. (DOCX 18 kb) [file 12862_2017_960_MOESM1_ESM.docx]

| **Population** | **No of individuals caught for CORT** | **Migration** | **Latitude**, **longitude and**  **altitude** | **No of nests** | **Clutch size:**  **mean ± se, (range)** | **Ref.** |
| --- | --- | --- | --- | --- | --- | --- |
| Ireland | 28 | partial | 51°49`N, 8°00`W, 21 m asl | NA,  6 | 5.0 ± 0.1 - 5.5 ± 0.1,  5.2 ± 0.8, (4 -6) | [1],  this study |
| Germany | 9 | short | 51°47`N, 6°01`E, 15 m asl | 237; | 5.2 ± 0.1, (3-7); | [2] |
| Central Spain | 24 | short | 40°32`N, 4°49`W, 1350 m asl | 19 | 5.2, (3-6) | [3] |
| Southern Spain | 28 | resident | 37°39`N, 5°34`W, 40 m asl | 38 | 4.2 ± 0.1, (3-5) | pers. comm. David Serrano |
| Canary Islands | 20 | resident | 28°46`N, 14°31`W, 57 – 359 asl | 128 | 2.7 ± 0.2 – 3.3 ± 0.1, (2-5) | [4] |
| Kenya, Mount Kenya | 9 | resident | 0°09’ N, 37°18’ E; 2269 asl | NA | NA | NA |
| Kenya, Olkalou | 9 | resident | 0°16’ N, 36°21’ E; 2416 asl | 1 | 3 | this study |
| Kenya,  Kinangop | 15 | resident | 0°37’ N, 36°29’ E; 2470 asl | 21 | 3.2 ± 0.1, (3-4) | this study |
| Tanzania, Ngorongoro | 13 | resident, altitudinal migrants?* | 3°08`S, 35°40`E; 2335 m asl | 1 | 2 | this study |
| Tanzania, Arusha National Park | 33 | resident | 3°16`S, 36°51`E; 1573 m asl | 8  102 | 2.8 ± 0.2, (2-3),  2.9 ± 0.06, (1-4) | this study,  pers. comm. Alexander Scheuerlein |
| Tanzania,  Monduli | 53 | resident | 3°14`S, 36°25`E; 1923 m asl | 30 | 2.8 ± 0.2, (2-4) | this study |
| Tanzania,  Usambara Mountains | 27 | resident | 4°30`S, 38°13`E; 1533 m asl | 5 | 2.8 ± 0.2, (2-3) | this study |
| Tanzania, Pare Mountains | 19 | resident | 4°18`S, 37°57`E; 1563 m asl | 5 | 2.8 ± 0.2, (2-3) | this study |

Supplementary material Table 1. Geographical and life history information of populations studied. No of nests: Number of nests on which clutch size estimate is based. Multiple entries in a field represent estimates from different studies; if only single studies are cited, multiple entries refer to different clutches or years, as specified in the table. Life history information of stonechats breeding in Southern and Central Spain is from a population close to the one that was sampled in our study and from a population in Catalunya (North East of Spain), respectively. * Based on one observation of one ringed individual during the nonbreeding season.

1. Cummins ST and O'Halloran J. The breeding biology of the stonechat Saxicola torquata in southwest Ireland. . Ir Birds. 2003;72:177-186.

2. Flinks H and Pfeifer F. Brutzeit, Gelegegröße und Bruterfolg beim Schwarzkehlchen (Saxicola torquata). Charadrius. 1987;23:128-140.

3. Muntaner J, Ferrer X, and Martínez-Vilalta A. Atles dels ocells nidificants de Catalunya i Andorra. Barcelona: Ketres Editora; 1983.

4. Illera JC and Díaz M. Reproduction in an endemic bird of a semiarid island: a food-mediated process. J Avian Biol. 2006;37:447-456.
